# Surgical residents' challenges with the acquisition of surgical skills in operating rooms: A qualitative study

**DOI:** 10.30476/jamp.2020.87464.1308

**Published:** 2021-01

**Authors:** LEILA SADATI, SHAHRAM YAZDANI, PEIGHAM HEIDARPOOR

**Affiliations:** 1 Virtual School of Medical Education and Management, Shahid Beheshti University of Medical Sciences, Tehran, Iran; 2 Department of Medical Education, Virtual School of Medical Education and Management, Shahid Beheshti University of Medical Sciences, Tehran, Iran; 3 Department of Community- Based Health Education, Virtual School of Medical Education and Management, Shahid Beheshti University of Medical Sciences, Tehran

**Keywords:** Skills, Education, Operating room, Qualitative research

## Abstract

**Introduction:**

Training in operating rooms is challenging. Specifically, surgical residents often experience a stressful environment in training arenas that, in turn, might affect their ability in the acquisition of the required qualifications. This study aims at the qualitative explanation of how the surgical residents acquire the surgical skills in operating rooms.

**Methods:**

This qualitative study was conducted in 2019-2020 using the conventional content analysis method. Participants were selected using purposive sampling. Data were collected through 25 semi-structured in-depth interviews. Then, the interview transcriptions were analyzed in MaxQDA2 software using the content analysis method.

**Results:**

The data were classified into two main categories, namely challenges/obstacles and strategies for dealing with the challenges. The data in the first category were further classified into four subcategories, including burnout, confusion in technique selection, unequal learning opportunities, ignorance, and responsibility misassignment. Similarly, four subcategories of establishing communication channels with chief residents and faculty members, learning by the non-surgeon pathway, covert progress in the learning path, and taking advantage of force from a position of power in the learning path were considered for the second category.

**Conclusion:**

Based on the findings of the study, surgical residents face serious challenges and obstacles in their training course. To address these challenges, the curriculum of the surgical course needs to be improved with the emphasis on the balanced responsibility assignment and enhanced human communication.

## Introduction

Surgical residents often experience a tense environment in training arenas ( 1 ). Their training in operating rooms faces several challenges, including a decline in academic records compared to the last century, demographic changes, economic constraints, and changes in attitudes toward surgery training, with an emphasis on patient safety ( 2 ). The number of open surgical procedures is declining, and they are abandoned in some cases, so the residents' opportunity to gain experience and practice has reduced or removed. On the other hand, the number of complex endoscopic and laparoscopic interventions is increasing ( 3 ). The introduction of less invasive technologies and, consequently, the possibility of different endoscopic interventions over the past two decades has challenged the training and evaluation of surgical residents ( 4 ). Research studies also show that some surgical residency graduates do not have the necessary qualifications ( 5 ). Researchers believe that traditional surgery training with senior/junior hierarchy in relationships cannot meet the need for learning complex technical skills in today's operating room, in a constrained period of residency training. The atmosphere of apprehension in the signor/junior residency training system for surgical residents causes rejection, resentment, and severe stress, which affect their learning ( 6 ). In 2019, the American Society of Surgeons emphasized the necessity of adjustments in this training environment. According to the statement, harassment, coercion, and discrimination are three distinct interpersonal behaviors that can negatively affect professional relationships, job satisfaction, and physical/mental health. In addition to threatening the health of employees, these behaviors create a hostile work environment that can endanger the safety of patients ( 7 ). Therefore, the improvement of this educational arena, development of constructive communication, and creation of an interactive educational environment are emphasized ( 8 ).

On the other hand, training courses and working hours of residents have been affected by the financial issues of hospitals and economic pressures prevailing in medical centers ( 9 ). As future health care practitioners, the better they are trained with opportune planning and practical training in a proper context, the more qualified employees would be to provide high-quality services ( 10 ). Over the past few decades, the development of structured and efficient approaches to surgical courses, including attention to setting primary educational goals, efficient evaluation methods based on receiving feedback, and the use of effective teaching models has been highly emphasized ( 11 ). In this regard, several educational models, including the Zwisch model, which provides both faculty and residents with specific stages of supervision, allow for adequate, safe training in a graduated manner to develop operative autonomy and fully trained surgeons. The other excellent model is Briefing-Intraoperative-Debriefing (BID) model that walks the learner through the surgery in three stages. During the briefing stage, the attending surgeon starts the conversation with a brief question about the goal of the operation or previous experiences. In the intraoperative stage, the attending surgeon will still coach and guide the resident through the operation and, finally, in the debriefing stage, the preceptor and learner debrief about the encounter. Also, assessment tools such as Objective Structured Assessment of Technical Skills (OSATS) are widely used to score the skills of each surgical trainee in performing or assisting in real operations. Similarly, Ottawa Surgical Competency Operating Room Evaluation (O-SCORE) is a 9-item surgical evaluation tool designed to assess technical competence in surgical trainees using behavioral anchors ( 12 - 15 ). 

However, in many operating rooms, surgical residency training is still performed in a challenging and stressful environment, based on the lecturer-student model in which residents must learn in a competitive situation through observation and by watching the performance of instructors or senior residents, performing the procedure under the supervision of senior residents, and eventually teaching junior residents during their residency course ( 16 ). In this learning framework, they try to fulfill the assigned responsibilities of patient safety by adopting coping strategies in different encounters and avoiding conflicts, and at the same time, improving their surgical skills ( 17 ). Since there was no accurate information on the challenges and strategies used by surgical residents in operating rooms, the present qualitative study was conducted through in-depth semi-structured interviews with the purpose of the explanation of surgical residents' experience in the acquisition of surgical skills.

## Methods

The present study is a qualitative, conventional content analysis with the purpose of explanation of surgical residents' experience in surgical skills acquisition in operating rooms.

The study settings were 11 academic hospitals in 8 cities of Iran, including Tehran, Karaj, Ardabil, Rasht, Qazvin, Tabriz, Shiraz, and Mashhad.

Resident participants in this research were selected based on the study purpose and maximum diversity in academic records, gender, age, and university/medical college where they were training. The inclusion criteria of the study were continuous participation in practical residency courses in the operating room for at least six months and consent to participate in interviews. Eligible participants entered the study after discussing the study objectives and oral consent. We determined the number of required respondents by interviewing the residents who met the inclusion criteria until the data were saturated, and no new topics were generated. The saturation was achieved at the 25th interview.

The research data were collected through in-depth semi-structured interviews with 25 surgical residents. The interviews were conducted in person in the operating rooms and residents' pavilion, or pre-planned online interviews through WhatsApp. 

The interviews began with a sample of the following general questions for a mean of 30 to 45 minutes per session.

- Explain your training day in the operating room?
- What is your opinion about the general surgery program?
- How do your training and evaluations direct you to higher levels?

Then, based on the participant's answers, provocative or exploratory questions were asked to deepen the interview. For example:

- Can you give us more details?
- Please give an objective example of what you just said.
- How did you feel then?

 After the 25th interview, the data was saturated, and no new information was added.

The data analysis process was performed using conventional content analysis methods in three phases: preparation, organization, and reporting ( 18 ). In the preparation phase, immediately after each interview session, the recorded content was accurately transcribed in detail and entered the MaxQDA software to facilitate the analysis process. Then, each interview was read several times during the organization phase. After repeated readings, semantic units related to the research question were selected, and the initial open codes were written for them. Three hundred ninety-eight open codes were extracted. Similar open codes were classified into a subcategory in terms of notion and meaning. Using constant comparison methods in the last stage of the data analysis, similar items were merged, and the main categories were identified to explain the surgical residents' experience in the acquisition of surgical skills in operating rooms.

Trustworthiness and Consistency: 

We used Cuba and Lincoln's five criteria of credibility, transferability, dependability, authenticity, and conformability to strengthen the data ( 19 ).

To verify the raw data, the transcribed interviews were sent to the participants via email or WhatsApp. Recording reminders and notes in the field, emphasis on sampling with maximum variability (age, sex, marital status, operating rooms in different colleges, different years of residency), and peer review were among the researchers’ measures to ensure the validity of the study. Undoubtedly, the researcher's disposition in teaching and research in the field of medical education and the operating room also contributed to the theoretical sensitivity and validity of the study.

### Ethical Considerations

The present research is based on the results of a doctoral dissertation approved by the ethics committee of Shahid Beheshti University of Medical Sciences with IR.SBMU.SME.REC.1398.057 moral license number.

With respect to principles of research ethics, all research participants gave their consent, they were assured their data would be kept confidential, and their audio files would be deleted at the end of the study. They were also assured that they were authorized to refrain from participation at any stage of the research process.

## Results

There were 25 participants in the study, including eighteen male residents and seven females. Five of them were in the first year, 5 in the second year, 6 in the third year, 6 in the fourth year, and 3 in the fifth year of their residency course. Sixteen residents were married, and nine were single. The mean age of the male and female participants was 36±3.2 and 32±2.3 years, respectively. 

The results of common content analysis related to surgical residents' experiences of the clinical training process were categorized into two main categories: challenges /obstacles and strategies for dealing with challenges (Table 1).

**Table 1:** The main extracted categories and subcategories

Main category	Subcategory
Challenges and Obstacles	Burnout
	Confusion in technique selection
	Unequal learning opportunities
	Neglect and disregard
	Responsibility misassignment
Strategies for dealing with challenges	establishing communication channels with chief residents and faculty members
	learning through the non-surgeon pathway
	covert progress in the learning path
	taking advantage of force from the position of power in the learning path

In the following section, the main extracted categories and subcategories are described. 

### Challenges and Obstacles

Five subcategories including burnout, confusion in technique selection, unequal learning opportunities, neglect and disregard, and responsibility misassignment constituted this main category. 

### A. Burnout

 High working pressure, imposition of irrelevant and humiliating roles, unnecessary physical presence, and assuming responsibility are primary codes in this subcategory. The heavy workload imposed on residents, especially in the first and second years, leads to extreme fatigue and burnout. 

"In the first year, there is excessive physical work and constant tiredness that leads to nervousness and weight loss up to 10 kg" (R27L1M).

In addition to excessive workload, assuming unrelated and humiliating tasks and the obligation to perform such assignments is one of the most common complaints of residents. 

"In the first year of residency, you have to do every banal and seemingly worthless task. It is as if they wanted to crush you. They call you and say, "Hey rookie, prep and transfer the patient". "Come here and implant a urinary catheter". While there are many other staff there, including the operating room team, to do so, it is just you that must go there and perform patient catheterization. It's really humiliating" (R8L2M).

Sometimes, despite the lack of assigned tasks for residents, they are not allowed to leave the hospital or even the operating room simply because other residents have not completed the operation. Thus, unnecessary physical presence was one of the issues that the residents complained about.

"Well, I think this is clearly a case of forced labor. It is unbearable for God's sake. I don't have anything else to do, but I have to go to the pavilion for nothing, while at least I can use this opportunity to get some rest or drop in my home" (R15L2M).

As the level of education of the residents increases, the physical burden decreases, but their responsibilities are added, and this is also a serious challenge to them.

"Just in the fourth year, the pressure of responsibility and challenges of decision-making get much worse; the responsibility of the patient ... other things, all are very bothersome. The system expects you, as a chief resident, to take the responsibility of a foreman, to coordinate, manage, and educate juniors" (R4L4M).

### B. Confusion in technique selection

Another challenge that assistants face with during their training course is the variety of techniques used by educators and senior residents, which can sometimes lead to incorrect learning of a procedure.

"In the case of performing a procedure, it's very rare that we can learn from a chief resident or educator. Well, first, we don't comprehend, but in the next years and by working as an assistant for senior residents, we gradually identify faults in previous training and try to correct them" (R28L3F).

They have to find a way to choose between these various techniques without questioning their senior residents.

"One of the problems is that the distance with the seniors is too much, and it may lead to incorrect learning. The seniors may not know how to do it well, or they may have learned it incorrectly and consequently teach you something wrong. You must check yourself frequently to make sure that what you have learned is correct. But you must do it indirectly because if seniors realize that you are checking them, it really makes them angry. Of course, there is disagreement even among the seniors, but finally, there will be a level of comprehension where one can come up with a choice of different methods" (R23L4M).

### C. Unequal learning opportunities

Learning opportunities, influenced by various factors, are not distributed equally. These factors are the dominance of relationships over criteria, incompatibility of the assistant to a patient, conflict of opportunity and threat due to the presence of fellowships, and gender discrimination. 

Due to the lack of a structured educational system for residency training, relationships are very influential in the training process of residents and repeatedly affect learning opportunities. 

"When somebody becomes the senior's or educator's favorite, she saves face and gets most of the attention by the educator; then, they don't pay attention to others, and even if she makes a mistake, she is treated very differently" (R12L2F).

The mismatch between the number and variety of patients and residents of different years is also a problem that inevitably affects their learning opportunities.

"There are too many residents, and you must have certain qualifications to get a procedure relevant to your education level. Generally, procedures with higher educational potential are for attendant residents. Less educational procedures remain for the rest, where you just handle the routines and complete unfinished tasks of seniors" (R8L2M).

The presence of laparoscopic fellowships has also led to a decrease in open surgery admittance, although their passion for teaching laparoscopic surgery principles is a great opportunity. 

"You know, some fellowships are very good-tempered and teach enthusiastically, but we always do the same things and just hold instruments for them. Well, if they weren't here, we might have more variety in procedures, and we would perform better in procedures while learning more things" (R19L3F).

In addition to the same problems as male residents, female residents disclosed the serious challenge of gender discrimination and ignorance of them by the educator in the operating room. 

"There is serious discrimination. We are underestimated. They don't count on us. They simply tell boys: 'Yes, we don't expect girls to keep up with us, you put yourself together. They pay more attention to boys and care more about them.' They see no good in girls, and they just use girls to fill circulatory shifts anyway" (R17L3F).

### D. Neglect and disregard

Deprivation of the right to choose and marginalization are primary codes to this class. 

Residents stated that they had been denied the right to choose and were mostly recruited as aids to finish operations, disregarding the correspondence between the type of operation and student's level and purposes of education. 

"Sometimes senior residents, instead of putting me on an appendectomy surgery, assign me to a laparoscopy procedure just to hold instruments while sending somebody else for that operation" (R25L1M).

Related to the challenge of being ignored, the marginalization and preventing juniors' comments were also mentioned by the residents. 

"The worst case is where a stubborn senior or educator gets angry with you. They act as if they did not see you anymore, and you won't get any procedure. You are just marginalized, and only God may help you in that situation" (R10L3M).

### Responsibility misassignment 

The heavy responsibilities assigned to juniors and the incompatibility of responsibilities and capabilities are classified into this subcategory. 

Unexpectedly and despite the low level of knowledge and skills in junior residents, visiting patients in the emergency department and performing surgeries in overnight shifts are among the serious responsibilities that have been assigned to them. Such a misassignment, according to some residents, leads to numerous and sometimes irreparable medical errors.

"Well, a first-year resident may visit a patient in the emergency room and order appendectomy inadvertently because of misdiagnosis. Such situations are frequent, and there are too many cases of misdiagnosis" (R5L2M).

On the other hand, when residents reach their final years of the course, despite a higher level of ability and skills, they spend most of their time resting in the pavilion and devote most of their time to studying to get better scores in Board Examination. This incompatibility between capabilities and responsibilities is evident in all levels and stages of residency training. 

"Now in our country this is upside down, one in 4th year of residency knows what to do, but does nothing, and one in the first year doesn't know what to do, and does everything. This is a long-standing tradition, and no one accepts to change it" (R24L4M).

### Strategies for dealing with challenges

Although there are many challenges in the field of education, residents are looking for different ways to acquire the necessary skills or to somehow pass this course and graduate. This main category consists of four subcategories, including establishing communication channels with chief residents and faculty members, learning through the non-surgeon pathway, progressing covertly in the learning path, and taking advantage of force from a position of power in the learning path.

### A. Establishing communication channels with chief residents and faculty members

This subcategory consists of primary codes, including trying to gain the trust of faculty members and create lobbies, and emulating successful chief residents. Because in most centers, residency training has been assigned to chief residents, one of the strategies that some residents adopt to gain experience and skills is to establish close relationships with chief residents and faculty members. Junior residents try to gain trust and to lobby in order to secure more learning opportunities or facilitate the promotion process. 

Residents believe that gaining the trust of educators in critical situations can be very effective in providing learning opportunities from faculty members. 

*"When the situation is a bit messy, if you can do it well and the educator understands that you are capable and you deserve it, he will hold your hand. It will give you a chance, and you will grow" (R9L3M)*.

Given the impact of informal communication on attracting the faculty members and senior residents' attention, one of the strategies mentioned by some residents is to try to create a lobby. 

"All you have to do is to try to become the professor's favorite one, and then you will be assigned as chief resident. Well, then you will have freedom of choice. Although it adds responsibilities, you can improve your surgery records."(R2L4M)

Another common strategy chosen by residents to establish a communication channel with faculty members and chief residents is to emulate successful senior or chief residents. They try to follow their path by modeling their performance.

"Sometimes, we observe the performance of the chief residents. We try to make them our role models and make progress" (R12L2F).

### B. Learning through the non-surgeon pathway

Learning from the members of the surgical team, particularly the scrub and circular nurse, was also a strategy that most residents mentioned to learn some basics of surgery and even performing minor operations, especially in the first and second years of residency. 

*"I think the scrub staff are key members who we need to learn from, especially for first-year residents who, I actually think, always must observe them and scan their attitude and performance. They must learn proper principles of prep and drape, how to deal with the equipment in the OR, the name of the equipment and instruments, the function of them, and many other things. I performed many surgeries with them. For example, an appendectomy is one of the operations that you see that actually scrub and circulating nurses know better than you, and they tell you what to do. Actually, they do the surgery better than you do" (R11L5M).* 

### C. Covert progress in the learning path

Taciturnity, avoiding participation in unrelated discussions, and observing the surgeons' hands during the training course are other approaches in this subcategory that residents choose to go through, as a challenging path. A group of residents believed that the best way to pass this course is to be quiet and avoid contention and questioning.

"Look, I just want to pass this course, and then I can apply for plastic surgery subspecialty. It doesn't matter if they teach me or not, I try to avoid verbal arguments and stay on the safe side, so that I can just graduate, that's all" (R15L2M).

Some residents believe that the cause of many communication challenges is the collateral issues that are made by some residents in different residency training groups, and they must be avoided. 

"Although what juniors conceive is almost correct, you can't be sure who's with or against you. It's better to stay away from collateral issues. There is a hierarchy here, and you must obey it anyway. After all, if they trust you, they will provide you with a good opportunity" (R20L4M).

Learning through observation, while others are being taught, is another covert learning strategy selected by residents.

"If you are an observer of a suitable surgery, where a chief resident is teaching a senior resident, you will learn a lot just by careful listening and watching. I learned many things when a chief was teaching a 4th-year resident" (R19L3F).

### D. Taking advantage of force from the position of power in the learning path

Performing the procedures suitable for junior residents on behalf of them (patient grab), assigning their own tasks to juniors in order to have the opportunity for more study, and choose the operation with the priority of his/her own learning needs are primary codes under this subcategory.

The use of force from a position of power in the residents was an issue that was noted on various occasions by the residents. Occasionally, some senior residents with lower experience in a certain surgery utilize their position to perform the surgery to increase their surgery records and skills, while actually that procedure is arranged for junior residents. 

"Well, I remember that when I was in the third year, our group had cholecystectomy surgery in the schedule, but one of the 4th-year residents didn't allow us to perform it because he wanted to gain more experience. Since there is no supervision, one in the position of power rules others" (R21L4M).

In some cases, senior residents leave their work and responsibilities to junior residents to stay in the pavilion and study for the board examination.

"Well, in the fourth year, you find an opportunity to delegate your tasks and responsibilities to junior residents and take your time to study more and get a better score in Board Examination. I know sometimes this is not correct, but it's the routine" (R23L4M).

Some residents also stated that senior residents always considered their own priorities in the scheduling of surgeries and arrange surgery plans for residents based on it. 

"Well, in the operating room, the chief resident provides the surgery plan and determines which surgeries are going to perform in which operating rooms. But the fact is that the chief resident selects whatever surgeries he likes for himself" (R12L2F).

## Discussion

The present qualitative study was conducted in 2019 to explain the experiences of surgical residents with field training in the operating room. Two main categories of challenges and strategies for dealing with challenges were extracted based on the analysis of conventional content analysis. 

The main category of the challenges included burnout, confusion in technique selection, unequal learning opportunities, neglect and disregard, and responsibility misassignment subcategories. Occupational burnout consisted of primary codes, including enduring the high and low-quality workload of juniors, imposing humiliating roles, and burnout. One issue that was mentioned repeatedly in many interviews was the burnout caused by irrelevant and humiliating tasks assigned to residents. These tasks were non-educational and were considered the duty of a practical nurse or other health care staff. Residents spend a lot of time performing tasks like recording and reporting, etc. which can also be done by non-physician staff ( 20 ). Based on other studies, job burnout is a syndrome of emotional fatigue and characterization that decreases the effectiveness of the workplace and should be managed ( 21 ).

Another category extracted under the challenges subcategory is the confusion in technique selection. Due to the variety of techniques used by senior residents or faculty members, junior residents are always in search of the right method. Postgraduate students, including surgical residents, are faced with a variety of surgical techniques and procedures by faculty members in everyday practice, and they must be able to adapt to it ( 22 ). The results of another study show that faculty members also confirm the technical diversity among the surgeons and consider it as a part of the surgical culture ( 23 ).

Some primary codes, such as the incompatibility of the assistant to a patient, confrontation with events and the threat of the presence of fellowships, and gender discrimination, constitute the subcategory of unequal learning opportunities.  The incompatibility of the assistance with the patient and educational goals and creation of a competitive environment have confronted the residents with serious challenges in acquiring surgical skills. Along with these results, Bradley believes that a structured curriculum should be designed to achieve educational goals, given the reduction in students' learning opportunities following the decline in situations for gaining experience ( 24 ). Despite the rare reports of positive academic interactions, the results of our study show that the presence of specialized fellowships in the operating room has led to a decrease in learning opportunities for residents. In line with the present study, the results of other studies also show that the presence of fellowships in ORs produces a competitive environment, where the residents are deprived of the opportunity to gain experience from senior/chief residents. Gender discrimination was another issue raised by female residents, depriving them of the opportunity to learn ( 25 ). In several studies, gender discrimination has been one of the effective factors in the training of surgical residents. Among these findings are the results of another study, which emphasizes the impact of female gender on learning opportunities and autonomy ( 26 ). In this regard, de Costa et al. also stated that males still outperform females in a number of procedures and sub-specialties, and the most important of which is surgery ( 27 ).

Another subcategory in this category was "being ignored". This subcategory is made up of primary codes, including the right to choose and marginalize. According to the results, residents did not have the right to select and were often marginalized, thus missing the learning opportunities. While confirming the prevailing atmosphere of intimidation and harassment in the training system of surgical residents, they stated that according to their study, during the residency course, more than 70% of surgical residents in Canada were harassed by senior residents with various means, including rejection and insult ( 28 ).

The last subcategory related to the category of challenges was the incompatibility between capabilities and responsibilities. The first- and second-year residents, who do not yet have sufficient qualifications in the diagnosis and treatment of diseases, are responsible for the admission and discharge of patients even though they are highly prone to diagnostic errors because of extreme fatigue resulting from multiple working shifts along with a low level of knowledge and skills. This happens while the senior and chief residents, as the residents in charge, spend most of their time in the pavilion studying for Board Examination despite relative or complete competence in diagnosing and managing diseases after gaining experience in their residency course. This mismatch between the competency and the delegated responsibility may threaten the health and safety of residents and patients. Although we couldn't find a similar study for comparison with the results of the present study, which can be due to the specificity of this situation for residency education programs in our country, undoubtedly, there was a significant relationship between the knowledge and skills of residents and other health care staff ( 29 ). Using evaluation based on Entrustable Professional Activities (EPAs), researchers noted the difference in the competence of residents at different levels ( 30 ). Therefore, it seems that assigning the responsibilities should be commensurate with the training level of the residents, so that they can provide the safest and highest quality care to patients with the least error according to their knowledge, experience, and competence.

 In the face of serious challenges and obstacles along the training course, residents develop different strategies. Establishing communication channels with senior residents and faculty members, learning through the non-surgeon pathway, progressing covertly in the learning path, and taking advantage of force from a position of power in training were subcategories that formed the category of "strategies for dealing with the challenges" in this study. In a qualitative study, on the impact of the governing hierarchy on the residency course, they pointed to the atmosphere of fear and intimidation surrounding the training environment in the operating room and reported the developed strategies by residents. These included acceptance of conditions and adaptation, avoidance of conflict, and fulfillment of assigned responsibilities to protect the patient ( 17 ). One of the solutions that residents choose to overcome the obstacles and challenges is to consider the senior residents and faculty members as their role models. They find that by carefully observing the performance and interactions of senior residents and surgeons, they can find an effective shortcut to success. In an exploratory study, the importance of faculty members' role as role models in job promotion and job selection for surgical residents was emphasized ( 31 ). Also, based on the results of this study, residents improved their learning in a strategy based on observing the hands of senior residents and faculty members. The researchers emphasize that many residents still learn the surgery by observing the hands of others and then imitate them ( 16 ). In order to continue their studies in fellowship, some residents are trying to pass this course by avoiding discussion and following the seniors' orders. Surgical residents try to apply for fellowships in various disciplines after completing a surgical residency course ( 32 ). Learning from non-surgeons was another strategy for dealing with challenges. They learn skills such as hand washing, instrument preparation and setting, working with instruments and equipment, and even performing small surgeries such as appendectomy from scrub and circulating nurses. Since in other countries the role of the scrub nurse as the surgeon's Senior Assistant is performed by other groups such as physicians' assistants, the influential role of physicians' assistants in the success of surgical residents is noted by reducing the working hours of residents ( 33 ).

Another subcategory extracted from the category of strategies for dealing with the challenges was the force from the position of power. In this strategy, the chief and senior residents, mostly if they have not accomplished full potential in some surgeries, perform the surgical procedures suitable for junior residents, and they will be deprived of these opportunities. This cycle is repeated for residents of different levels. Seizing the learning opportunities by senior residents is also mentioned in another study ( 3 ). Stewart Gabel points to the "power in leadership" and explains the utilization of power and its effects on interactions between managers and subordinates ( 34 ). The results of several studies show that common issues such as lack of clear learning objectives, fear, anxiety, humiliation and intimidation, prevention from participation in surgery, and limited learning opportunities have been effective barriers to the satisfaction of medical training groups in the operating rooms ( 35 ).

### Limitation 

The major limitation of the present study was the inability of researchers to travel to other cities for data collection and in-person interviews. The researchers tried to address this limitation by arranging extensive online interviews.

## Conclusion

Based on the findings of the study, the lack of a structured training program and dominance of the senior/junior system cause the surgical residents to face serious challenges and obstacles in their training course. This situation sometimes threatens their physical and mental health. However, over time they develop strategies such as establishing communication channels to get through this course, even if they fail to acquire the necessary skills. Therefore, the researchers suggest that authorities in charge of developing the surgical education system take serious steps to improve the curriculum of the surgical course and monitor the systematic implementation of these changes. 
